# Correlation between lipid metabolism levels and pregnancy outcomes

**DOI:** 10.3389/fmed.2025.1530525

**Published:** 2025-02-12

**Authors:** Jingjing Guo, Haifan Qiu, Jianping Wang, Xiaowei Liu, Suipeng Chen, Baoqing Li

**Affiliations:** ^1^Department of Laboratory Medicine, The Second Affiliated Hospital of Wenzhou Medical University, Wenzhou, China; ^2^Department of Obstetrics and Gynecology, The Second Affiliated Hospital of Wenzhou Medical University, Wenzhou, China

**Keywords:** adverse pregnancy outcome, interval of reference, lipid metabolism, parturient, pregnancy

## Abstract

**Objective:**

To establish the reference interval for the serum lipid index in pregnant women and to explore the relationship between lipid metabolism levels and pregnancy outcomes.

**Design and methods:**

Data were derived from 446 pregnancy women and 317 healthy non-pregnant women. Serum levels of total cholesterol (TC), triglycerides (TG), high-density lipoprotein cholesterol (HDL-C), low-density lipoprotein cholesterol (LDL-C), apolipoprotein A1 (ApoA1), apolipoprotein B (ApoB), lipoprotein (a) [Lp(a)], and hypersensitive C-reactive protein (hs-CRP) were measured in both groups. The mean and standard deviation of each index were calculated to establish the reference range of normal serum lipid levels in pregnant women in mid-to-late pregnancy. The associations between serum lipid levels and perinatal outcomes were assessed statistically.

**Results:**

There were no significant differences in age, pregnancy, or parity between the adverse outcome and normal delivery groups, but the caesarean section rate was significantly higher in the adverse outcome group. The levels of hs-CRP, TG, TC, HDL-C, LDL-C, and ApoA1 were significantly higher in the adverse outcome group. Elevated hs-CRP, TG, and HDL-C levels were risk factors for adverse pregnancy outcomes. According to the receiver operating characteristic curve, the optimal threshold of the combined diagnosis of these three indicators to predict adverse pregnancy outcomes was 0.534, and the area under the curve was 0.822.

**Conclusion:**

The establishment of lipid reference intervals in the second and third trimesters of pregnancy can effectively evaluate lipid metabolism in pregnant women, and the measurement of lipid metabolism in pregnant women is helpful in predicting adverse pregnancy outcomes.

## 1 Introduction

Lipids are crucial bioactive components that maintain the growth and development of the embryo and placenta. In clinical practice, lipid metabolism indicators primarily include total cholesterol (TC), triglycerides (TG), high-density lipoprotein cholesterol (HDL-C), low-density lipoprotein cholesterol (LDL-C), apolipoprotein A1 (ApoA1), apolipoprotein B (ApoB), and lipoprotein (a) [Lp(a)]. To ensure normal foetal growth and development and maintenance of pregnancy in the second and third trimesters, a physiological state of hyperlipidaemia occurs in the blood; however, this is pathologically insignificant (1, 2). While for some individuals, lipid levels can be excessively elevated, transitioning from a physiological to a pathological state. Such exacerbated hyperlipidaemia can have detrimental effects on foetal health and may pose risks to children’s long-term cardiovascular health. Evidence confirms that cholesterol levels in pregnancy are positively correlated with blood pressure even 6 and 9 years postpartum, and lead to an increased risk of long-term cardiovascular disease, threatening subsequent health (3).

Nevertheless, there is currently no standardised reference interval for serum lipid levels during the second and third trimesters, either domestically or internationally. Current reference values in China were derived from the “Chinese Adult Blood Abnormality Prevention and Treatment Guidelines” published in 2016. However, these guidelines do not accurately evaluate the blood concentration levels specific to pregnant women. The absence of consensual criteria to distinguish between pathologic and physiologic changes in maternal lipid metabolism is a significant barrier to the clinical identification of hyperlipidaemia during pregnancy and subsequent intervention and treatment. Huang et al. (4) proposed reference ranges of lipid levels in pregnancy by the mean and standard deviation to determine appropriate percentiles, while Zhu et al. (5) estimated reference intervals by the receiver operating characteristic curves.

Pregnancy involves changes in certain metabolic processes that lead to increased blood concentration levels within a certain range. However, exceeding the normal limit can increase viscosity, leading to excessive accumulation on the uterine wall with potential damage (6), which may result in conditions such as preeclampsia or gestational diabetes mellitus (7). Studies have shown that abnormal lipid metabolism during pregnancy and delivery can lead to adverse outcomes (8). However, the risk assessment of adverse pregnancy outcomes is unclear because of differences in the metabolic mechanisms of serum lipid indicators. Evidence confirms that women with higher triglycerides, sensitivity CRP, and lower HDL-C were more likely to develop hypertension post-delivery (9). Konrad et al. (10) showed that a serum Lp(a) level >40.5 mg/dl in a mildly preeclamptic patient predicted the development of severe preeclampsia later on in the pregnancy. A comprehensive understanding of how variations in serum lipid levels affect pregnancy outcomes is vital. Our study aimed to establish reference intervals of lipid indicators suitable for the Wenzhou region. A secondary aim was to explore the relationship between different lipid metabolism indicators and pregnancy outcomes by analysing the lipid metabolism levels of pregnant women.

## 2 Materials and methods

### 2.1 Study targets

A reference interval was established: 446 pregnant women in the second and third trimesters of pregnancy who visited our hospital from July to December 2019 were included in the study. Inclusion criteria were local women in mid-to-late pregnancy (13–41 weeks) documented in our hospital with complete data, women aged 18–48 years old with singleton pregnancies, and those who consumed a normal diet, were non-smokers, and had no drug or alcohol intake. Exclusion criteria were a previous history of hypertension or diabetes and those with gestational diabetes mellitus, gestational hypertension disease, or a macrosomia. Simultaneously, 317 healthy nonpregnant women of childbearing age were selected as controls.

For a correlation study, 91 pregnant women with adverse pregnancy outcomes who gave birth at our hospital from September to November 2020 were collected as the adverse pregnancy outcome group, and 83 pregnant women without adverse pregnancy outcomes who gave birth at our hospital during the same period were collected as the control group. The inclusion criteria were women aged 18–48 years old with singleton pregnancies who gave birth in our hospital and had complete data records. None of the pregnant women were taking drugs that would affect serum lipid metabolism. The exclusion criteria were a previous history of hypertension and diabetes, patients with severe liver, kidney, endocrine, or circulatory system diseases or metabolic insufficiency, those with twin or multiple pregnancies or who underwent assisted reproduction surgery. Adverse pregnancy outcomes included premature delivery, dystocia, stillbirth, macrosomia, foetal distress, placental membrane-related problems, and neonatal asphyxia.

### 2.2 Specimen detection methods

All subjects were required to fast for approximately 12 h. Venous blood (WS/T463-2015, Health Industry Standard of the People’s Republic of China) was collected in the early morning of the next day, biochemical vacuum tubes (BD, lot No. 0019774) were placed, and serum was separated after blood coagulation. The German Cobas C501 automatic biochemical analyser was used to detect serum lipid-related items, in which TC, TG, HDL-C, and LDL-C were original reagents from Roche; ApoA1 and ApoB were reagents from Siemens of Germany; Lp(a) was produced by Shanghai Fuxing; and hypersensitive C-reactive protein (hs-CRP) was produced by Aurea of Finland.

Reference interval verification and establishment of the test results of 20 pregnant women (reference discharge criteria) were randomly selected to verify the current reference interval of serum lipids. If no more than two cases of each test item fell outside the reference interval, the reference interval verification was effective. If more than two cases were identified, an additional 20 pregnant women were selected for re-verification based on the aforementioned criteria. Should there still be more than two cases after re-verification, it indicates that the current reference interval is inadequate and a new reference interval must be established (11). The reference interval for the serum lipid index of pregnant women in this area was established using a normality test after collecting all serum lipid indices and eliminating outliers.

### 2.3 Statistical analysis

#### 2.3.1 The outlier test adopted the Dixon method

First, the test results were arranged in order of magnitude, the range (R) was calculated, and then the difference (D) between the maximum and minimum values and their adjacent values was calculated. If D/R ≥ 1/3, the maximum or minimum value was considered as an outlier and removed. The preceding steps were repeated for the remaining data to perform the outlier test until all outliers were eliminated.

#### 2.3.2 Statistical processing

SPSS Statistics 26.0 software was used for analysis. The measurement data of normal distribution were presented as x̄ ± SD, and the measurement data of skew distribution were presented as *M* (*P*25–*P*75). The *t*-test or rank-sum test was used for comparison between the groups. A logistic regression model was used to determine the risk factors leading to adverse pregnancy outcomes, and the receiver operating characteristic (ROC) curve was used to analyse its value in predicting adverse pregnancy outcomes. *P* < 0.05 was defined as statistically significant.

## 3 Results

### 3.1 Verification of current reference interval of serum lipid indices of pregnant women in Wenzhou

By randomly selecting the test results of 20 pregnant women to verify the current reference intervals of serum lipids, we found that the reference intervals of eight adult serum lipids were not applicable to pregnant women (Table 1). Therefore, it is necessary to establish a reference interval for serum lipids suitable for pregnant women in Wenzhou.

**TABLE 1:** Verification of the reference interval of eight lipid indices in pregnant women in the second and third trimesters.

	Original reference intervals	Number of matches	Number of mismatches	Non-conformance rate (%)
hs-CRP	0.0–3.00 mg/L	15	5	25
TG	0.5–1.70 mmol/L	3	17	85
TC	<5.18 mmol/L	2	18	90
HDL-C	≥1.04 mmol/L	15	5	25
LDL-C	<3.37 mmol/L	4	16	80
ApoA1	1.20–1.60 g/L	1	19	95
ApoB	0.8–1.20 g/L	5	15	75
Lp(a)	0–300 mg/L	15	5	25

hs-CRP, hypersensitive C-reactive protein; TG, triglyceride; TC, total cholesterol; HDL-C/LDL-C, high-density/low-density lipoprotein cholesterol; ApoA1, apolipoprotein A1; ApoB, apolipoprotein B; Lp(a), lipoprotein (a).

### 3.2 Establishment and validation of reference intervals for serum lipid indices in pregnant women from this region

The reference intervals for serum lipid indices were established and validated according to the health industry standard WS/T402-2012 of the People’s Republic of China. Outliers were excluded as per protocol. Normality tests revealed that the TC, HDL-C, LDL-C, ApoA1, and ApoB levels in both the pregnancy and control groups followed a normal distribution. The reference interval was determined using x ± 1.96S approach. The TG, Lp(a), and hs-CRP levels exhibited skewed distributions; hence, a 95% reference interval was established using percentiles (*P* 2.5, *P* 97.5). Subsequently, these reference intervals for the second and third trimester were successfully verified (Table 2).

**TABLE 2:** Verification results of reference interval in the second and third trimester.

	Second trimester				Third trimester			
	**Reference intervals**	**Range of 20 individuals**	**Number of mismatches**	**Verification result**	**Reference intervals**	**Range of 20 individuals**	**Number of mismatches**	**Verification result**
hs-CRP (mg/L)	0–11.93	2.85–8.17	0	Pass	0–10.16	3.41–7.58	0	Pass
TG (mmol/L)	1.23–5.56	2.25–4.89	0	Pass	1.69–5.70	2.05–4.74	0	Pass
TC (mmol/L)	3.92–7.64	4.20–7.45	0	Pass	3.98–8.48	4.23–8.25	0	Pass
HDL-C (mmol/L)	1.13–2.55	1.33–2.46	0	Pass	1.01–2.47	1.74–2.37	0	Pass
LDL-C (mmol/L)	1.60–5.12	1.84–4.55	0	Pass	1.53–5.85	1.69–5.16	0	Pass
ApoA1 (g/L)	1.64–2.66	1.78–2.39	0	Pass	1.54–2.76	2.15–2.61	0	Pass
APOB (g/L)	0.61–1.43	0.92–1.22	0	Pass	0.61–1.67	0.94–1.53	0	Pass
Lp(a) (mg/L)	0–598	37–430	0	Pass	0–567	79–516	0	Pass

hs-CRP, hypersensitive C-reactive protein; TG, triglycerides; TC, total cholesterol; HDL-C/LDL-C, high-density/low-density lipoprotein cholesterol; ApoA1, apolipoprotein A1; ApoB, apolipoprotein B; Lp(a), lipoprotein (a).

### 3.3 Comparison of basic clinical data and serum lipid levels between the two groups

Among the 174 participants, 91 were in the adverse outcome group (52.3%) and 83 were in the normal delivery group (47.7%). There were no significant differences in mean age, body mass index (BMI), birth history, or age distribution between the adverse outcome group and the normal delivery group (*P* > 0.05). However, the rate of caesarean section in the adverse outcome group was significantly higher than that in the normal delivery group (*P* < 0.05). We further compared the serum lipid levels of the two groups and found that the hs-CRP, TG, TC, HDL-C, LDL-C, and ApoA1 levels in the adverse outcome group were higher than those in the normal delivery group, with statistical significance (*P* < 0.05). There were no significant differences in ApoB and LP(a) between the two groups (*P* > 0.05) (Table 3).

**TABLE 3:** Comparison of basic clinical data and serum lipid levels between the adverse outcome group and the normal delivery group.

	Total	Adverse outcomes group	Normal delivery group	*P*
Number	174	91	83	
Age	29 (26–32)	29 (27–32)	28 (26–31)	0.743
BMI	26.33 (24.36–28.34)	26.44 (24.65–28.63)	25.98 (24.27–27.54)	0.112
Birth history	Multiparous	35 (38.47)	39 (46.99)	0.256
	Primiparous	56 (61.53)	44 (53.01)	
Age distribution	≥35	12(13.19)	9 (10.84)	0.636
	<35	79(86.81)	74 (89.16)	
Delivery mode	Normal labour	64 (70.32)	75 (90.36)	0.001
	Caesarean birth	27 (29.68)	8 (9.64)	
hs-CRP (mg/L)		4.07 (2.30–11.95)	2.89 (1.72–4.33)	0.000
TG (mmol/L)		3.17 (2.51–3.84)	2.79 (2.06–3.16)	0.000
TC (mmol/L)		6.28 ± 1.12	5.65 ± 0.89	0.000
HDL-C (mmol/L)		1.85 ± 0.33	1.69 ± 0.28	0.001
LDL-C (mmol/L)		3.67 ± 1.01	3.26 ± 0.80	0.004
ApoA1 (g/L)		1.98 ± 0.31	1.84 ± 0.29	0.002
ApoB (g/L)		1.14 ± 0.29	1.5 ± 0.25	0.839
Lp(a) (mg/L)		165.00 (74.0–343.0)	117 (60.00–247)	0.111

BMI, body mass index; hs-CRP, hypersensitive C-reactive protein; TG, triglycerides; TC, total cholesterol; HDL-C/LDL-C, high-density/low-density lipoprotein cholesterol; ApoA1, apolipoprotein A1; ApoB, apolipoprotein B; Lp(a), lipoprotein (a).

### 3.4 Logistic regression analysis of different lipid indices and adverse pregnancy outcomes

With the occurrence of adverse pregnancy outcomes as dependent variables and age, BMI, hs-CRP, TG, TC, HDL-C, LDL-C, and ApoA1 as independent variables, a binary logistic regression analysis was performed. The results showed that hs-CRP, TG, and HDL-C levels were risk factors for adverse pregnancy outcomes. The odds ratios were 1.266, 2.758, and 5.216, respectively (*P* < 0.05) (Table 4).

**TABLE 4:** Logistic regression analysis of different indicators and adverse pregnancy outcomes.

Characteristics	β	OR	95% CI	*P*
Age	0.035	1.036	0.941–1.14	0.471
BMI	0.134	1.143	0.984–1.328	0.081
hs-CRP (mg/L)	0.236	1.266	1.138–1.408	0
TG (mmol/L)	1.014	2.758	1.612–4.717	0
TC (mmol/L)	0.454	1.574	0.926–2.676	0.094
HDL-C (mmol/L)	1.652	5.216	1.265–21.5	0.022
LDL-C (mmol/L)	−0.025	0.975	0.551–1.725	0.931
ApoA1 (g/L)	0.723	2.061	0.492–8.64	0.322

β, partial regression coefficient; OR, odds ratio; CI, confidence interval; BMI, body mass index; hs-CRP, hypersensitive C-reactive protein; TG, triglycerides; TC, total cholesterol; TG, triglycerides; HDL-C/LDL-C, high-density/low-density lipoprotein cholesterol; ApoA1, apolipoprotein A1.

### 3.5 Value of different indicators in predicting and diagnosing adverse pregnancy outcomes

**FIGURE 1:**
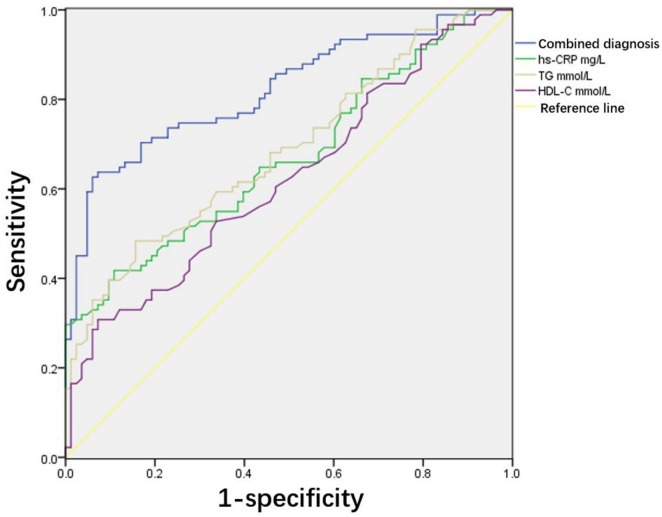
Receiver operating characteristic analysis of predictive value of serum lipids for adverse pregnancy outcomes. Area under the curve (AUC) of combined diagnostic indicators is 0.822 and 95% confidence interval (CI) is 0.76–0.833. The cut-off value of combined diagnostic indicators for predicting adverse pregnancy outcomes was 0.534. Sensitivity and specificity are 70.3% and 83.1%, respectively.

Taking the occurrence of adverse pregnancy outcomes as the diagnostic threshold, this study analysed the predictive value of hs-CRP, TG, HDL-C, and the combined diagnostic indicators composed of these three indicators. According to the ROC curve, the best threshold of combined diagnostic indicators for predicting adverse pregnancy outcomes was 0.534, sensitivity was 70.3%, specificity was 83.1%, and area under the curve was 0.822 (95% CI, 0.76–0.833). This was significantly higher than that predicted using a single indicator of adverse pregnancy outcomes (Table 5 and Figure 1).

**TABLE 5:** Value of different indicators in predicting and diagnosing adverse pregnancy outcomes.

	AUC	Cut-off value	Sensitivity	Specificity
Combined diagnosis	0.822	0.534	0.703	0.831
hs-CRP	0.671	0.257 mg/L	0.462	0.795
TG	0.687	0.327 mmol/L	0.484	0.843
HDL-C	0.623	0.236 mmol/L	0.308	0.692

AUC, area under the curve; hs-CRP, hypersensitive C-reactive protein; TG, triglycerides; HDL-C, high-density lipid cholesterol.

## 4 Discussion

As a special group, pregnant women experience physiological changes in lipid metabolism, including the accumulation of body fat tissue and an increase in liver lipid synthesis, which leads to an increase in lipid indices (1, 12). In the second and third trimesters of pregnancy, lipid metabolism changes further, with enhanced intestinal absorption capacity of fatty acids, decreased activity of liver lipase, enhanced fat decomposition capacity, increased production of fatty acids and glycerol, and increased liver synthesis of TG and other lipids, forming a physiological state of hyperlipidaemia. Woollett (13) also reported that lipid levels gradually increase in the second and third trimesters of pregnancy, and the placental lipid transport rate increases significantly, reaching a peak in the third trimester, which is a physiological phenomenon adapted to pregnancy. The physiological hyperlipidaemic state of pregnant women during normal pregnancy can meet the needs of their own physiological factors and foetal growth and development without pathological significance (14, 15). Currently, there is no unified standard for the normal reference interval for lipid levels in the third trimester of pregnancy either at local or worldwide levels. The current reference interval of lipid levels in China is taken from the “Guidelines for the Prevention and Treatment of Dyslipidaemia in Chinese Adults” published by the Center for Cardiovascular Disease Prevention and Control of the Ministry of Health in 2016 (16), where the recommended levels of TG, TC, HDL, and LDL are <1.7, <5.2, >1.0, and <3.4 mmol/L respectively. The Expert Suggestions on Improving the Rate of Clinical Lipid Control published in 2010 clearly indicate that the current reporting method for serum lipid test results (the reference interval is fixed) cannot meet the requirements of different treatment targets for patients with different risks (17). Therefore, this guideline does not provide an accurate standard for serum lipid levels in pregnant women. In addition, China is a vast country with different diets and living habits in different regions, so it is necessary to establish the serum lipid reference intervals specific to each region. The present study confirmed this by randomly selecting the test results of 20 pregnant women to verify the current serum lipid reference interval. The eight reference intervals for normal adult serum lipid levels that are currently used are not applicable to pregnant women. However, all items of the serum lipid reference interval established in this study for pregnant women in the second and third trimesters of pregnancy in this region have been verified.

Dyslipidaemia during pregnancy is closely related to adverse pregnancy outcomes, such as placental dysfunction, foetal distress, macrosomia, premature delivery, abortion, or foetal death (15, 18). Women with higher triglycerides, sensitivity CRP, and lower HDL-C were more likely to develop hypertension post-delivery (9). Singh et al. (19) showed preeclamptic women had elevations in fasting lipid profiles in the third trimester of pregnancy. Recent studies have found that elevated TG levels lead to vascular endothelial dysfunction, and enhanced lipid peroxidation leads to vascular endothelial cell damage (20). In normal pregnancy, the activity of the antioxidant system is simultaneously increased to resist lipid peroxidation, and endothelium-dependent relaxation is enhanced to protect the cardiovascular system. In addition, HDL-C protects vascular endothelial cells by removing fat from tissues. Therefore, abnormal lipid peroxidation does not occur during normal pregnancy. The general theory is that relatively low HDL and high TG levels promote pathological changes in vascular pathology and placental tissue ultrastructure. However, in this study, we found that TG, HDL, and ApoA1 levels were higher in the adverse pregnancy outcome group than in the normal delivery group, and that TG and HDL-C were risk factors for adverse pregnancy outcomes. Although this seems counterintuitive, recent studies have reported similar results. Studies have shown that TC, TG, HDL-C, and LDL-C levels are associated with preterm birth (21). A study on the relationship between lipid metabolism and small-for-gestational-age infants found that elevated maternal HDL-C and LDL-C levels in the third trimester were risk factors for small-for-gestational-age infants, while high cholesterol levels in the third trimester were negatively associated with small-for-gestational-age infants. HDL-C levels were considered a risk factor for small-for-gestational-age infants (22). Misra et al. (23) also reported an inverse relationship between HDL-C levels and birth weight at all time points, starting at 10 weeks of gestation in overweight or obese women. In the non-pregnant population, HDL-C levels have a protective effect against cardiovascular diseases. However, in a diseased state, normal HDL is transformed into dysfunctional HDL, which changes the regulation of vascular endothelial cells. In future studies, we plan to investigate the relationship between HDL-C levels and pregnancy outcomes.

Owing to the lack of a suitable reference intervals for pregnant women, serum lipid detection during pregnancy is not routinely performed in the clinic, resulting in failure to detect pathological hyperlipidaemia during pregnancy in a timely manner. However, studies have shown that hyperlipidaemia is a risk factor for preeclampsia (24, 25). First, LDL-C is susceptible to oxidation and acts as an atherogenic agent. Atherosclerotic factors can damage vascular endothelial cells, thereby inducing vasospasm (26). Elevated levels of TC and TG contribute to endothelial dysfunction. A decrease in vasodilatory substances such as prostacyclin leads to an imbalance with vasoconstrictive substances like thromboxane A2, triggering vasospasm and resulting in the occurrence of preeclampsia (27). The reduction in the vascular protective factor HDL-C weakens the anti-atherosclerotic effect in pregnant women, placing the body in a decompensated state. It is evident that lipid metabolic disturbances exacerbate atherosclerosis, enhance oxidative stress, and subsequently damage endothelial cells. Additionally, abnormal lipid levels reduce nitric oxide synthesis, leading to vasoconstriction and diastolic dysfunction, which trigger systemic small artery spasm and abnormally high blood pressure, thereby providing conditions for the development of hypertensive disorders of pregnancy (HDP) (28). Thus, abnormal lipid metabolism can enhance oxidative stress and inflammatory responses and finally cause vascular endothelial cell injury and dysfunction, which is involved in the pathophysiological process of hypertensive disorders during pregnancy (29). Hypertensive disorders during pregnancy are associated with serious adverse pregnancy outcomes (30). Therefore, understanding the relationship between maternal serum lipid levels and adverse pregnancy outcomes and studying the predictive value of timely intervention for high-risk maternal patients with dyslipidaemia during pregnancy may improve adverse pregnancy outcomes in this population. Some researchers have found that the mid-trimester TG level is an independent predictor of neonatal birth weight (31). Jin et al. (20) showed that pregnant women with hyperlipidaemia had a higher incidence of macrosomia. In this study, the predictive values of hs-CRP, TG, and HDL-C and the combined diagnostic indices of these three indicators were analysed. The ROC curve showed that the combined diagnostic indices were significantly better than the single indices in predicting adverse pregnancy outcomes. In the future, we will study the relationship between serum lipid levels in early, middle, and late pregnancy and adverse pregnancy outcomes. We hope to identify high-risk pregnant women with abnormal serum lipids during pregnancy at an early stage, implement timely management through diet and lifestyle, and improve the pregnancy outcomes of this population.

## 5 Conclusion

In summary, serum lipid reference intervals for pregnant women in the second and third trimesters of pregnancy in this region were established in this study, and all items were verified. We also found that the lipid metabolism levels in pregnant women were related to premature delivery, macrosomia, foetal distress, and other adverse pregnancy outcomes. hs-CRP, TG, and HDL-C levels are risk factors for adverse pregnancy outcomes, and their combined diagnosis is helpful in predicting the occurrence of adverse pregnancy outcomes. Appropriate clinical intervention should be carried out to reduce the occurrence of adverse maternal and infant outcomes and protect maternal and infant health.

## Data Availability

The datasets presented in this study can be found in online repositories. The names of the repository/repositories and accession number(s) can be found in this article/supplementary material.
